# Effect of RSK4 on biological characteristics of colorectal cancer

**DOI:** 10.1186/s12957-018-1474-7

**Published:** 2018-12-22

**Authors:** Qingqing Ye, Xuan Wang, Min Jin, Meng Wang, Yan Hu, Shihu Yu, Yonghua Yang, Jiyuan Yang, Jun Cai

**Affiliations:** 1 Department of Breast Surgery, First Affiliated Hospital of Yangtze University, Jingzhou, 434000 Hubei China; 2 Department of Oncology, First Affiliated Hospital of Yangtze University, Jingzhou, 434000 Hubei China; 3 Cancer Center, Union Hospital, Tongji Medical College, Huazhong University of Science and Technology (HUST), Wuhan, Hubei China

**Keywords:** RSK4, Colorectal cancer, Tumor suppressor gene, Epithelial–mesenchymal transition, E-cadherin

## Abstract

**Background:**

This study aimed to investigate the expression of P90 Ribosomal Protein S6 kinase 4 (RSK4) in colorectal cancer cells and its biological function.

**Methods:**

We selected early SW480 and HCT116 colorectal cancer cell lines, using Lipofectamine™ 2000 transfection reagent carrying RSK4 gene transfected into cells to establish the colorectal cancer cell lines with high expression of RSK4. RT-PCR and western blot (WB) analysis confirmed RSK4 expression in SW480 and HCT116 cancer cell lines. We used methylthiazoltetrazolium (MTT) assay and flow cytometry to detect the proliferation of colorectal cancer cells. After transfection of RSK4, the effect of RSK4 on the RNA levels associated with epithelial–mesenchymal transition (EMT) of colorectal cancer cells was analyzed by real-time fluorescence quantitative PCR and the expression of EMT-related protein was detected by WB analysis.

**Results:**

After transfection of RSK4 overexpression, the MTT assay detected that RSK4 could significantly inhibit the growth of colorectal cancer cells in vitro; flow cytometry detected that S-phase cells decreased significantly, and G0/1 cells increased significantly (*P* < 0.05). The invasion ability of SW480 and HCT116 cells transfected with RSK4 was markedly lower than that in the control group, and the difference was statistically significant (*P* < 0.05). Fluorescent quantitative PCR and WB analysis showed that the expression of EMT-associated molecular E-cadherin was remarkably increased and the expression of Snail was significantly decreased (*P* < 0.01).

**Conclusion:**

RSK4 gene in colorectal cancer cell lines with low expression of RSK4 after transfection can inhibit the growth and invasion of tumor cells. RSK4 gene may inhibit EMT and inhibit metastasis of colorectal cancer cells, may be a potential tumor suppressor gene and inhibit tumor distant metastasis, and may provide the biological basis for new therapeutic targets.

## Background

Colorectal cancer is the most common malignant tumor among gastrointestinal tract cancer. The distant metastasis of colorectal cancer cells is closely related to the morbidity and mortality of the disease. Further study of the pathogenesis of colorectal cancer is urgently being explored, to discover more effective methods for prevention, diagnosis, and treatment of the progression of cancer. RSK4 is a recent discovery gene located on X chromosome of linkage genes [1]. RSK is the downstream target of Raf-MEK-ERK signaling pathways, which belongs to the serine threonine kinase family. So far, four RSK members have been found in this family, They were play an important role in various life activities such as regulating cell proliferation, movement, growth, survival, and invasion.

The literature has reported that RSK4 gene can inhibit different breast cancer cell line growth, invasion, apoptosis, and metastasis both in vitro and in vivo [2–7]. Although it may be a suppressor gene of cancer, there are opposite reports that exogenous RSK4 gene had no obvious inhibitory effect on breast cancer progress [8]. Dewdney et al reported RSK4 expression in normal endometrium, and that the expression is lower or missing in endometrial carcinoma [9]. RSK4 is highly methylated in endometrial carcinoma cell lines and endometrial carcinoma compared with normal endometrium. It is not certain currently whether RSK4 is a tumor suppressor gene for endometrial cancer. Fan et al. and another 16 studies have found that RSK4 plays an important role in the prognosis of renal cell carcinoma [2]. What role does RSK4 play in the end? Sun et al. [10] carried out a series of studies on the genes which can encode RSK4, suggesting that RSK4 is an oncogene or tumor suppressor gene depending on many factors. In our previous study, we have demonstrated that RSK4 expression was low in colorectal cancer, and immunohistochemical analysis found that the expression of RSK4 protein in colorectal cancer is closely related to the clinical pathology of lymph node metastasis, tumor grade, and clinical stage [11]. Therefore, we will have a deep discussion to clarify RSK4 gene play a role in the proliferation and invasion of colorectal cancer cells.

## Methods

### Cell culture

Human colorectal cancer cell lines SW480 and HCT116 are held by our unit. They were cultured in RPMI 1640 medium containing 10% fetal bovine serum (FBS) and cultured in an incubator containing 5% CO_2_ and 95% humidity. The culture medium was changed every 2 or 3 days depending on the state of cells, and the cells were passaged when they grew well and the density was between 80 and 90%.

### Transient cell transfection plasmid

Plasmid was extracted with a plasmid extraction kit without endotoxin. Transfection was carried out following the instructions of Lipofectamine™ 2000. A density of 5 × 10^5^ cells was inoculated into the six-hole plate 1 day before transfection. The cells were transfected with 4 μg plasmid and 10 μl Lipofectamine™ 2000 reagent; the control group was not transfected, and the negative control group was transfected with empty plasmid. After 4–6 h, the intact medium containing FBS was changed to continue culture.

### RT-PCR

The mRNA expression of RSK4 and EMT-related molecules in the SW480 and HCT116 cell lines respectively was detected by RT-PCR. Trizol was used to extract total cellular RNA, 3 μg total RNA for each cell. cDNA first-strand synthesis was performed by reverse transcription according to the reverse transcription kit. Primers were designed using Primer 5 software according to the gene sequences reported by GenBank and the literature. Each cell was subjected to three independent repeated trials. Finally, we took the average value of the three experiments. The primer sequences are presented in Table 1.

**Table 1:** Sequence of primers including EMT-related molecules, RSK4, and β-actin

Molecule	Primer
RSK4 forward	AATACTACCATTCGCTCCTCA
RSK4 reverse	TTACAGGCCAGTTGATGTTCGC
H-actin forward	GTCCACCGCAAATGCTTCTA
H-actin reverse	TGCTGTCACCTTCACCGTTC
H-CLDN-2 forward	GGCTTTCCACAGAGAGACGGG
H-CLDN-2 reverse	CAAGCAGCCTCAAGAAGGCAT
H-E-cadherin forward	CTTTGACGCCGAGAGCTAC
H-E-cadherin reverse	TTTGAATCGGGTGTCGAGGG
H-P53 forward	CCTCCTCAGCATCTTATCCG
H-P53 reverse	TTCCGTCCCAGTAGATTACCA
H-Snail forward	AGGACACATTAGAACTCACACGG
H-Snail reverse	CGAGTAAACATTGATTGCGTCAC
H-TGF-β forward	TACCTGAACCCGTGTTGCTCT
H-TGF-β reverse	TTTCCCCTCCACGGCTCAAC
H-twist forward	TGGCCTGCAAAACCATAGTCA
H-twist reverse	AGACACCGGATCTATTTGCAT
H-Vimentin forward	TCAGAATATGAAGGAGGAAATGGC
H-Vimentin reverse	GAGTGGGTATCAACCAGAGGGAGT

*EMT* epithelial–mesenchymal transition, *RSK4* P90 Ribosomal Protein S6 kinase 4, *TGF-β* transforming growth factor beta

### Western blot analysis

Detection of the protein level of RSK4 and EMT-related molecules in colorectal cancer cell lines SW480 and HCT116 was by WB analysis. RIPA pyrolysis liquid was used to extract protein from the cells 72 h after transfection, following the operating procedures in accordance with the instructions for RIPA lysate. The BCA method was used for measuring the protein concentration. After electrophoresis, the protein was transferred to the PVDF membrane, with 5% dried skimmed milk powder as a blocking reagent, mouse monoclonal antibody (1:2000), HRP- Goat Anti-Rabbit Secondary Antibody (1:4000). Before adding ECL luminous liquid and subsequent exposure in a floating bath.

### Methylthiazoltetrazolium assay

MTT assay was used to detect the growth of cells before and after transfection. The exponential growth of cells (nontransfection group, blank plasmid group, and transfected group) was inoculated in the culture dish. After 12, 24, 48, 72, and 96 h, the absorbance at 490 nm was measured by a microplate reader, and the killing rate of the cells was calculated.

### Flow cytometry to detect the cell cycle

Trypsin was used to digest cells in logarithmic growth. A sample of 10^6^ cells was suspended in PBS in a single cell suspension liquid. Cells were then fixed with ethanol precooled to − 20 °C, kept at 4 °C overnight, centrifuged at 2000 r/min, and washed twice with PBS. Flow cytometry was used to analyze the cell cycle after adding propidium iodide (PI) staining solution and incubating for 30 min at 37 °C.

### Transwell invasion assay

BD Matrigel was melted at 4 °C, and the medium and transwell plate were precooled. Then 100 μl diluted Matrigel was added into each upper transwell chamber, and incubated at 37 °C. The cells were collected and washed twice, and then suspended and counted, and the cells were diluted to 1 × 10^6^/ml. The remaining medium was discarded and remaining matrix gum was washed away with DMEM. Then 600 μl medium containing 10% FBS was added into the transwell chamber underneath. A 100 μl dilution of cell suspension liquid was added into the upper chamber. We cultured cells for 36–48 h at 37 °C with 5% CO_2_, washed with PBS, fixed with methanol, and dyed with crystal violet, washing off excess dyeing solution with tap water. Dyeing with crystal violet, the cells were mounted and viewed under a camera.

### Statistical methods

Student’s *t* test was used to examine the differences in cell activity, gene transcription level, or protein level expression in each group. Results were shown as mean ± standard deviation (mean + SEM). There was significant statistical difference when *P* < 0.05.

## Results

### Overexpression of RSK4 and detection of mRNA and protein in colon cancer cells

**Fig. 1:**
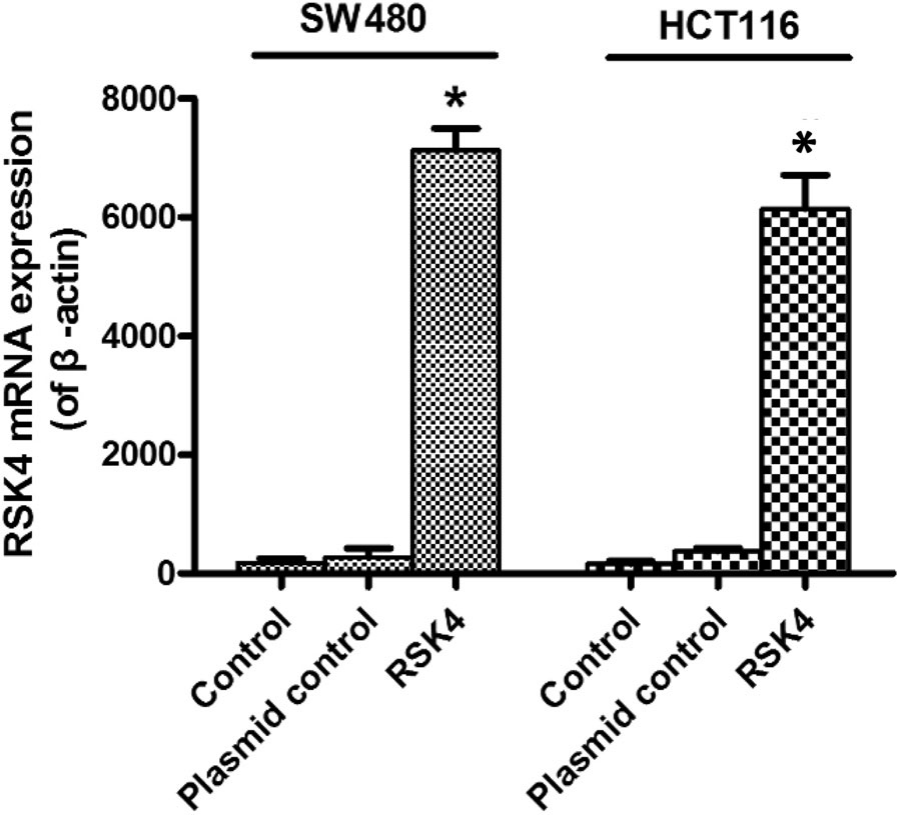
Validation of transfection efficiency in SW480 and HCT116 cells. mRNA expression level in blank control group, empty plasmid group, and transfected RSK4 gene group detected by RT-PCR. Expression of RSK4 mRNA detected after 48 h (*P* < 0.05). RSK4 P90 Ribosomal Protein S6 kinase 4

**Fig. 2:**
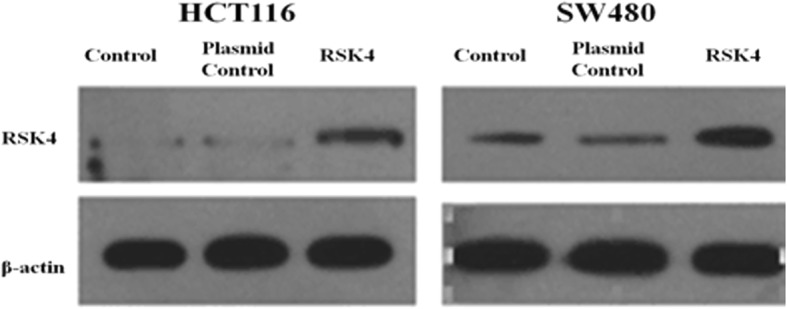
Validation of RSK4 transfection efficiency in SW480 and HCT116 cells. Expression level in blank control group, empty plasmid group, and transfected RSK4 gene group detected by WB analysis. Expression of RSK4 protein detected after 48 h (*P < 0.05). RSK4 P90 Ribosomal Protein S6 kinase 4

Our previous studies have shown low expression of RSK4 protein in colorectal cancer patients. We constructed RSK4 overexpression plasmid and transfected RSK4 gene into colorectal cancer cell lines (SW480 and HCT116). After 48 h, the transfection efficiency was verified by real-time PCR. The results showed that, compared with normal cells and the blank plasmid group, the expression level of RSK4 in the RSK4 transfected groups increased in magnitude and the difference was statistically significant (Fig. 1). WB analysis showed SW480 and HCT116 transfected with blank plasmid with shallow protein bands, suggesting low expression of RSK4 protein. The color of these bands became deep when transfected with overexpression plasmid, suggesting that protein expression increased after RSK4 transfection (Fig. 2).

### Overexpression of RSK4 could inhibit the growth of colorectal cancer cells

**Fig. 3:**
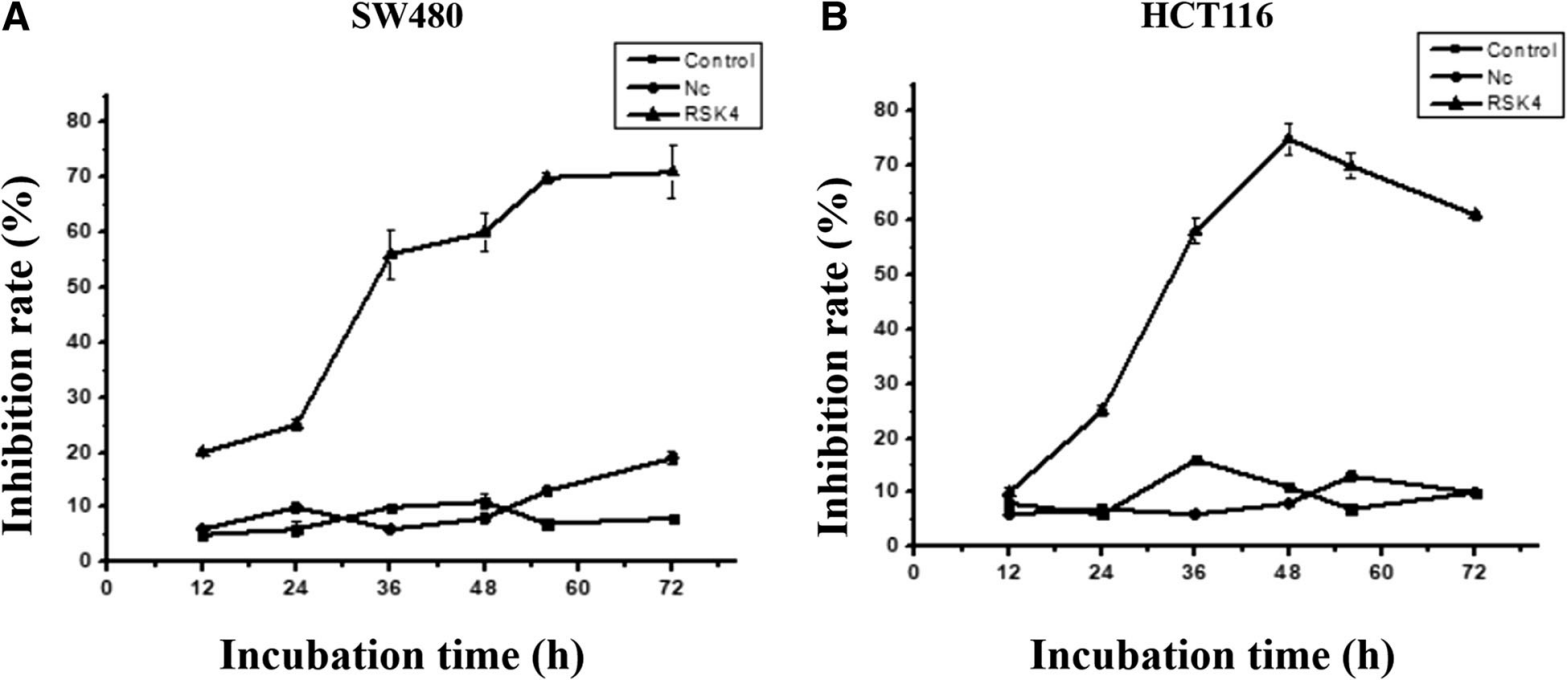
SW480 cell (**a**) and HCT116 cell (**b**) inhibition rate after transfection of RSK4. OD value detected at 12, 24, 36, 48, and 72 h. There was significant difference between control group and experimental group (*P < 0.05). RSK4 P90 Ribosomal Protein S6 kinase 4

In order to confirm whether RSK4 of colorectal cancer cells has a growth inhibiting effect in vitro, we transfected RSK4 gene into SW480 cells (Fig. 3a) and HCT116 cells (Fig. 3b). The OD value was detected in the blank control group, empty plasmid group, and transfected RSK4 gene group by the MTT method at 12, 24, 36, 48, and 72 h. The cell inhibition rate is equal to the blank group OD value minus that of the experimental group divided by the OD value of the blank group and multiplied by 100%. The growth curve showed that overexpression of RSK4 can inhibit the growth of colorectal cancer cells in vitro, the inhibition at 48 and 60 h was about 60–70%, while the normal group and blank plasmid group was about 10%; the difference has statistical significance (*P* < 0.05) and the late suppression effect reached a plateau stage.

### Effects of RSK4 overexpression on the DNA synthesis cycle of colon cancer cells

**Fig. 4:**
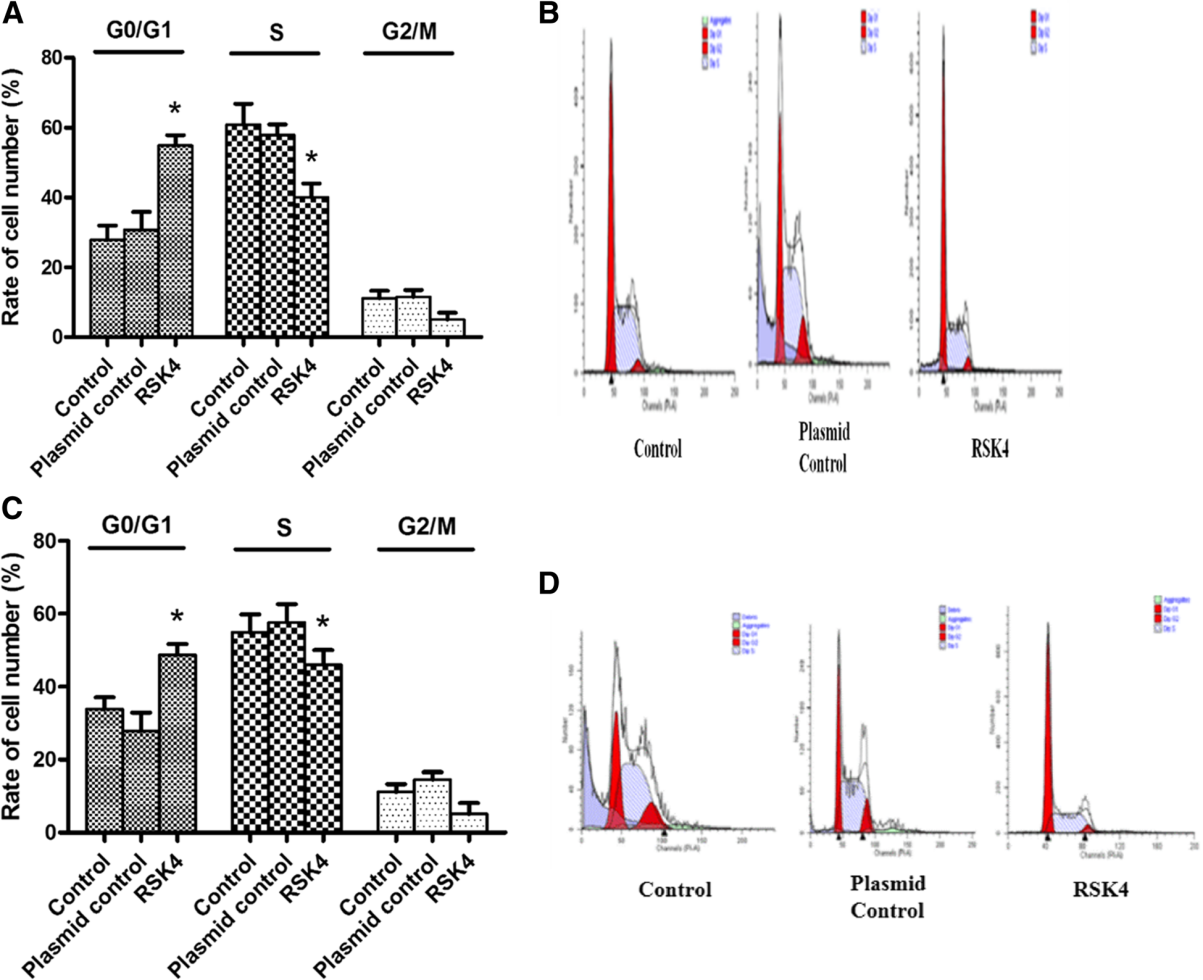
Effect of RSK4 on cell cycle. SW480 cells (**a, b**) and HCT116 cells (**c, d**) in blank group and transfected empty plasmid group. Transfected RSK4 gene cells cultured for 72 h, propidium iodide staining for cell cycle analysis. Percentage in S phase of each group decreased and percentage of cells in G0/1 phase increased. The difference was statistically significant (*P < 0.05). RSK4 P90 Ribosomal Protein S6 kinase 4

In order to further analyze the effect of RSK4 on the growth cycle of colorectal cancer cells, we used flow cytometry to analyze the cell cycle. The normal cells transfected with blank plasmid cells and transfected with RSK4 gene cells were cultured for 72 h and stained with propidium iodide (PI). We can detect the strength of DNA binding PI in cells by flow cytometry, which contributes to confirming the distribution of the cell cycle. The results showed that after transfection of RSK4, the percentage of S-phase cells among SW480 and HCT116 cells decreased, and the cells in the G0/1 phase increased obviously; the difference was statistically significant (*P < 0.05) (Fig. 4a–d) (Table 2).

**Table 2:** Change of cell cycle after RSK4 transfection in SW480 and HCT16 cells

Group	G0/G1 phase	S phase	G2/M phase
SW480 cell cycle (%)			
Control	27.91 ± 4.1	60.91 ± 5.9	11.17 ± 2.1
Plasmid control	30.82 ± 5.1	57.64 ± 3.0	11.54 ± 2.0
RSK4	54.9 ± 3.0	40.05 ± 4.0	5.05 ± 2.0
HCT 116 cell cycle (%)			
Control	33.91 ± 3.2	54.91 ± 4.9	11.17 ± 2.1
Plasmid control	27.82 ± 5.1	57.64 ± 5.0	14.54 ± 2.0
RSK4	48.8 ± 3.0	46.1 ± 4.0	5.1 ± 3.0

Change of cell cycle was detected 72 h after RSK4 transfection among different groups in SW480 and HCT16 cells (*n* = 3, mean ± standard deviation)

*RSK4* P90 Ribosomal Protein S6 kinase 4

### Effects of RSK4 overexpression on invasion and metastasis of colorectal cancer cell

**Fig. 5:**
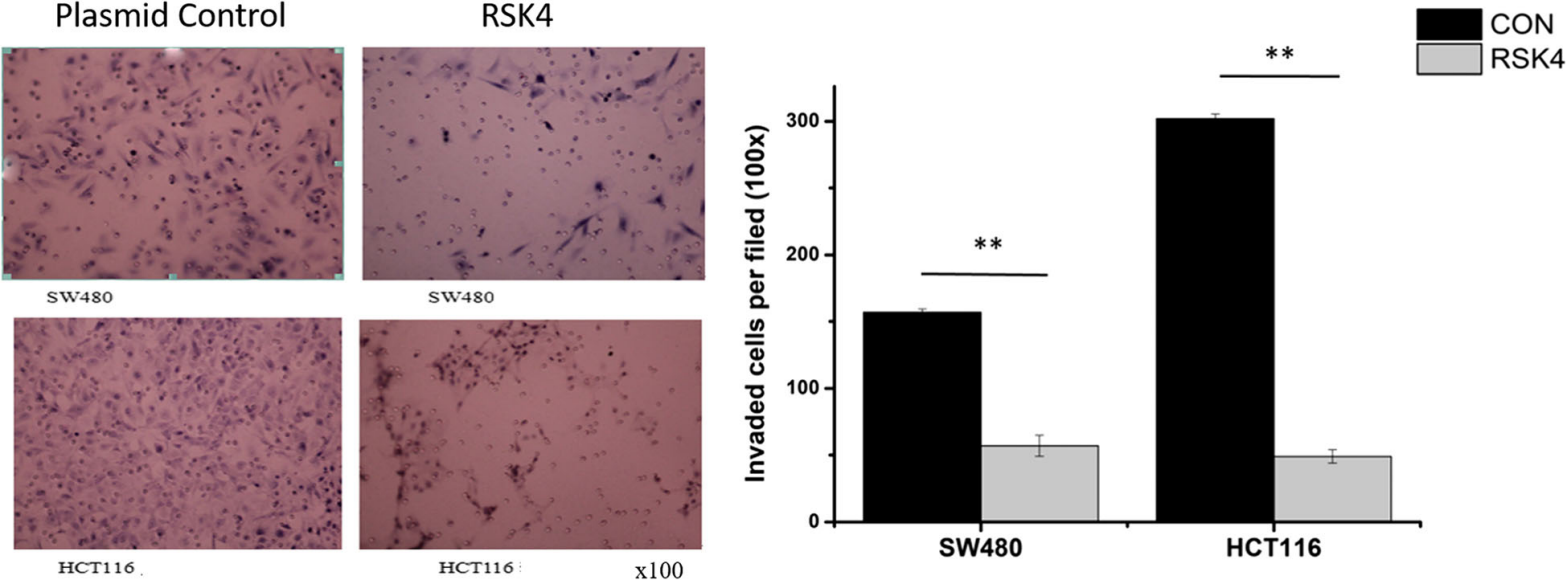
Detection of the invasion ability of cells transfected with RSK4 or Control (**P < 0.01)

The discussed experiments revealed the effect of RSK4 on the growth and proliferation of colorectal cancer cells in various aspects. Therefore, can RSK4 affect the invasion ability of colorectal cancer cells in vitro? A transwell invasion assay showed that the invasion ability of transfection of RSK4 in SW480 cells and in HCT116 cells was significantly lower than in the control group; the difference was statistically significant (Fig. 5). These phenomena show that overexpression of RSK4 can reduce the invasiveness of colorectal cancer cells.

### The effect of RSK4 on the expression of target gene mRNA

According to a large number of reports, RSK4 plays an important role in tumor invasion and metastasis, and tumor invasion and metastasis are closely related to epithelial–mesenchymal transition of tumor. Therefore, is the progress of RSK4 in tumor suppression associated with epithelial–mesenchymal transition? In order to determine whether RSK4 regulates these EMT-related genes, our first step was detection of the expression of mRNA (Claudin-2, E-cadherin, P53, Snail, TGF-β, Twist, and Vimentin) in the SW480 and HCT116 normal groups, empty plasmid group, and transfected RSK4 group by real-time quantitative PCR.

**Fig. 6:**
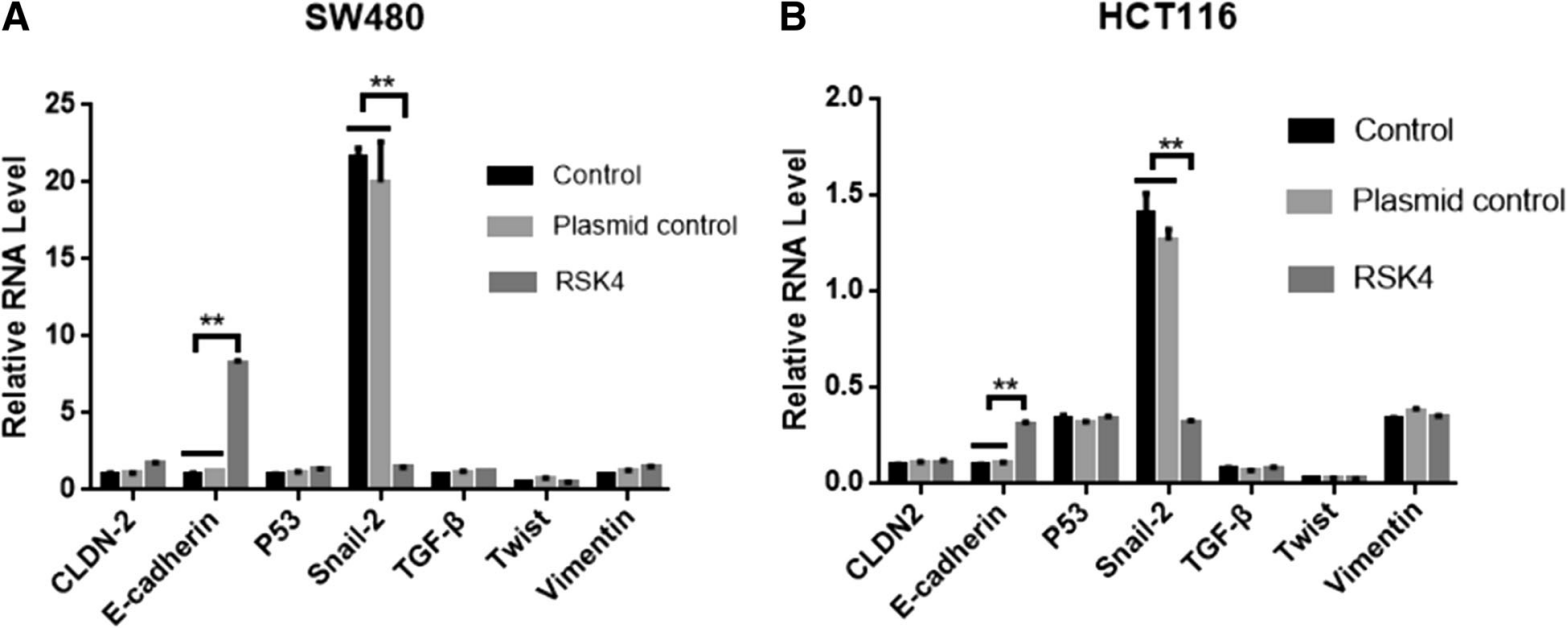
Real-time quantitative PCR analysis of effect of RSK4 on EMT-related molecule mRNA in SW480 cells (**a**) and HCT116 cells (**b**). Expression of E-cadherin increased significantly, and expression of Snail decreased significantly (**P < 0.01). RSK4 P90 Ribosomal Protein S6 kinase 4, TGF-β transforming growth factor beta

In the SW480 and HCT116 cell lines, we found high expression of E-cadherin in the transfected RSK4 group, especially in the HCT116 cell line at up to 14 times. The SW480 and HCT116 cell lines showed low expression of Snail, while Claudin-2, P53, TGF-β, Twist, and Vimentin mRNA expression levels did not change (Fig. 6a, b). These results suggest that RSK4 may play an important role in tumor invasion, metastasis, and epithelial–mesenchymal transition by regulating E-cadherin and Snail.

### Effect of the expression level of RSK4 on the protein level

**Fig. 7:**
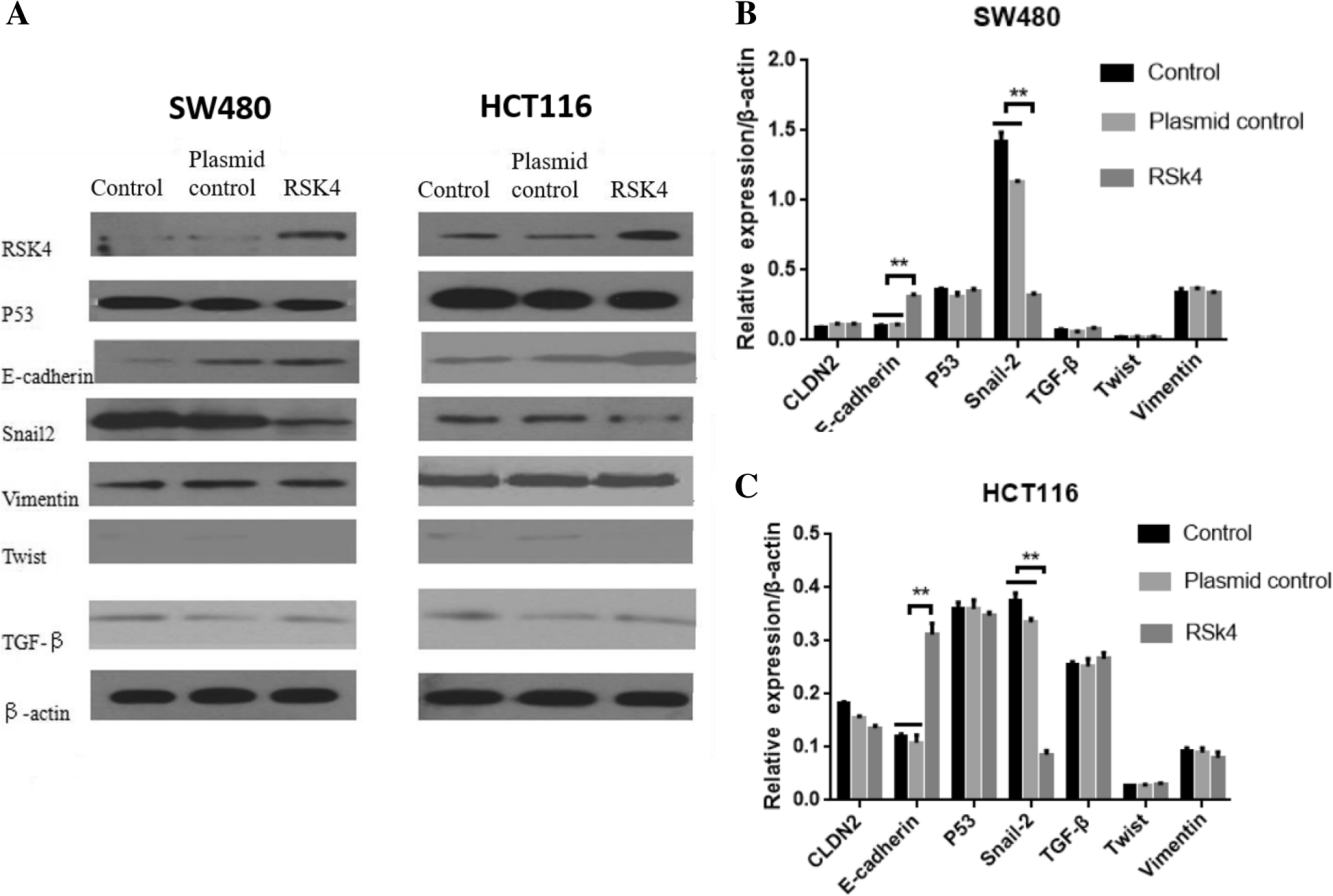
**a** WB analysis of effect of RSK4 on SW480 cells and HCT116 cells of EMT-related protein. Expression of EMT-related molecular protein after transfection of RSK4 gene in SW480 cells (**b**) and HCT116 cells (**c**). Expression of E-cadherin significantly increased, expression of Snail significantly decreased (**P < 0.01). RSK4 P90 Ribosomal Protein S6 kinase 4, TGF-β transforming growth factor beta

We also examined the effects of RSK4 on the translation of genes (Claudin-2, E-cadherin, P53, Snail, TGF-β, Twist, and Vimentin) by western blot analysis. We again showed high expression of E-cadherin protein in the transfected RSK4 group, and low expression of Snail protein in the SW480 and HCT116 cell lines, while the protein levels of Claudin-2, P53, TGF-β, Twist, and Vimentin did not change (Fig. 7a–c). This result is consistent with the results of the PCR analysis.

## Discussion

The incidence of colorectal cancer is increasing year by year in developed countries and developing countries. It is the third most common tumor in the world, and the mortality rate is fourth in the world [12]. In recent years, although the operation has made considerable progress, radiotherapy and chemotherapy need to be constantly updated. Various detection methods, biotherapy and targeted therapies for colorectal cancer are emerging, but without a revolutionary improvement, and they did not reach the expected results with a low 5-year survival rate. How to select specific molecular targets to attack tumors has become a difficult problem in current studies. Although bevacizumab and cetuximab achieved certain efficacy, they all encountered bottlenecks [13–17]. Finding a new target for colorectal cancer is imminent [18].

The Raf–MEK–ERK signaling pathway is a hot spot in colorectal cancer research. P90 ribosomal S6 Kinase (RSK) belongs to the serine kinase family, and is an important downstream target gene of the Raf–MEK–ERK signaling pathway. RSK4 is a linkage gene located on the X chromosome [19], and overexpression of RSK3 or RSK4 supports proliferation upon PI3K inhibition both in vitro and in vivo [20]. The X chromosome-linked oncogenes and tumor suppressor genes are regulated at the individual gene level. RSK4 inhibits the transcriptional activation of specific targets by effects on ERK, thereby inhibiting cell growth. Lopez-Vicente et al. [21] reported that expression of RSK4 decreased in colon cancer and renal cell carcinoma. At the subcellular level, RSK1–3 was found in the cytoplasm of quiescent cells, and a considerable part of the protein can be transferred to the nucleus if it receives stimulation. RSK4 mainly settles in the cytoplasm, and not in the nucleus from the stimulus of mitogen. The basic and clinical research of RSK4 in colorectal cancer is low, and only three papers have been reported at home and abroad.

Our previous studies have shown that RSK4 protein is highly expressed in colorectal cancer tissues, but low expressed in colorectal tissues. RSK4 may be one of the important biomarkers for the early diagnosis, prognosis, and treatment of colorectal cancer [22]. We used RT-PCR to detect the RSK4 expression level in SW480 and HCT116 cells, and the results showed that RSK4 expression was low or undetectable in these two cell lines. After optimizing the conditions, the plasmid was successfully transfected into the cells, and the RNA was extracted after culture for 48 h in a 37 °C incubator. We transfected RSK4 into different colorectal cancer cell lines, and then explored the specific biological function of RSK4 in colorectal cancer. In 48–60 h of transfection of RSK4 gene, the inhibition rate of SW480 cells was about 70%, while for the normal cells and blank plasmid group it was about 10%; the difference was statistically significant, while the late suppression effect reached a plateau. In HCT116 cells, the cells grew slowly after transfection of RSK4 gene, and the inhibition rate reached about 60% after 60 h of culture, while the normal cells and blank plasmid groups grew well, indicating that overexpression of RSK4 can inhibit the growth of colorectal cancer cells in vitro. For the sake of further analysis of the effect of RSK4 gene on the cell cycle of colorectal cancer, the cell cycle was detected by flow cytometry. The results showed the cell ratio of S-phase in normal cells and cells transfected with blank plasmid at around 57%. After RSK4 gene transfection, SW480 cells and HCT116 cells in the S phase significantly decreased to 40%. After transfection of RSK4 gene, G0/G1 phase cells increased significantly, and the difference was also statistically significant (*P* < 0.05). These results indicate that RSK4 gene inhibits tumor cell growth by inhibiting cell protein synthesis and may be a potential tumor suppressor gene.

Studies have shown that RSK4 also negatively regulates receptor tyrosine kinase (RTK) signaling during embryogenesis, inhibiting Ras–ERK signal transduction and cell proliferation [7, 23], so RSK4 with very low expression in the most rapid growth cells, upregulating intracellular expression, can inhibit cell growth, similar to our study. Thakur et al found that the expression of exogenous RSK4 had no effect on the growth of breast cancer cells [8]. But there are reports of the opposite; abnormal expression of some X-linked genes including RSK4 was found in breast cancer [23], indicating that abnormal RSK4 has a carcinogenic effect. Dewdney et al have reported RSK4 expression in the normal endometrium, and the expression is lower or missing in endometrial carcinoma, compared with normal endometrium [9]. It is not clear that RSK4 hypermethylation is an anti-oncogene in endometrial cancer. Sun et al performed a series of studies on the genes encoding RSK4, suggesting that RSK is an oncogene or tumor suppressor gene, depending on many factors [10].

## Conclusion

RSK4 is an X-linked gene. Although its function is not fully understood, there is a large amount of data showing that RSK4 is highly expressed in cancer cells which can reduce cell proliferation and mitosis and promote apoptosis [20, 24]. RSK4 is involved in multiple signaling pathways in cancer development. Therefore, the application of RSK4 in cancer therapy may have potential breakthroughs and become a new therapeutic target worthy of further study. 

## Abbreviations

EMT: Epithelial–mesenchymal transition
FBS: Fetal bovine serum
MTT: Methylthiazoltetrazolium
PI: Propidium iodide
RSK4: P90 Ribosomal Protein S6 kinase 4
RTK: Receptor tyrosine kinase
